# Tuning photocatalytic activity of g-C_3_N_4_ through Cu deposition *via* chemical reduction and a DBD plasma method for visible-light-driven Cr(vi) reduction

**DOI:** 10.1039/d5ra08483k

**Published:** 2026-03-31

**Authors:** Jana Petrović, Andjelika Bjelajac, Tihana Mudrinić, Jérôme Guillot, Simon Bulou, Rada Petrović

**Affiliations:** a Innovation Center of the Faculty of Technology and Metallurgy, Ltd Belgrade Serbia; b Luxembourg Institute of Science and Technology, Advanced Plasma and Vapor Deposition Processes Engineering L-4362 Esch-sur-Alzette Luxembourg; c University of Belgrade – Institute of Chemistry, Technology and Metallurgy – National Institute of the Republic of Serbia Belgrade Serbia; d University of Belgrade, Faculty of Technology and Metallurgy Belgrade Serbia

## Abstract

The dispersion of metal nanoparticles (NPs) on graphitic carbon nitride, g-C_3_N_4_ (CN) offers a promising strategy to improve its photocatalytic efficiency. In this work, Cu deposition on CN was attempted by wet chemical reduction with NaBH_4_ (Cu-CN-cr) and an atmospheric pressure dielectric barrier discharge (AP-DBD) cold plasma method (Cu-CN-pl). The samples were evaluated for Cr(vi) photocatalytic reduction under visible light at pH = 3, and compared with pristine CN and plasma-treated CN (CN-pl). FESEM, TEM, XPS and EPR showed that the Cu NPs were not formed by chemical reduction, but Cu^+^ was incorporated into the CN structure in the Cu-CN-cr sample. This caused a slight red shift of the absorption edge, reduced the PL peak intensity, and increased the photocurrent. In the Cu-CN-pl sample, Cu was deposited on the surface as Cu_2_O/Cu(OH)_2_ NPs, with the possibility of metallic Cu present within the core of the NPs. The plasma treatment induced remarkable structural modifications that served as binding sites for the NPs. The photogenerated electrons in the Cu-CN-cr were probably consumed for the reduction of Cu^+^ instead of Cr(vi), leading to a decrease in the photocatalytic reduction of Cr(vi). The use of plasma in the synthesis of Cu-CN-pl provided a uniform distribution and intimate contact between the deposited NPs and CN, thereby constructing an effective heterojunction. This heterojunction facilitates the separation of the carriers and enhances the photocatalytic activity compared to CN.

## Abstract 2

The dispersion of metal nanoparticles (NPs) on graphitic carbon nitride, g-C_3_N_4_ (CN) offers a promising strategy to improve its photocatalytic efficiency.

## Graphical abstract

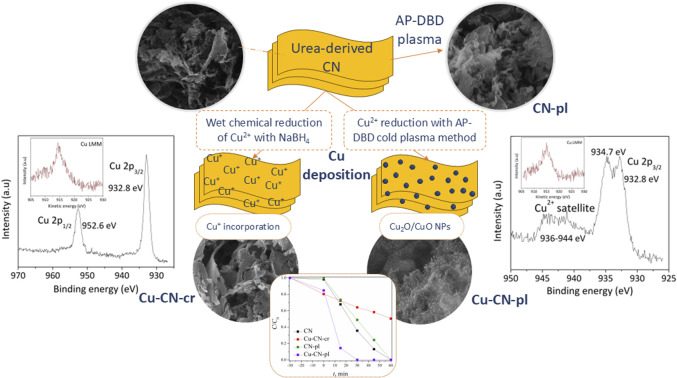

## Introduction

Hexavalent chromium (Cr(vi)) is a particularly problematic pollutant due to its widespread application, high toxicity, carcinogenicity, and mobility in aquatic environments.^1,2^ In order to protect human health and the environment, Cr(vi) in wastewater is most often chemically reduced to the less harmful Cr(iii), which is typically conducted at pH ≈ 2. Low pH value is beneficial for effective Cr(vi) reduction since the reduction rate of Cr(vi) increases at lower pH levels due to the higher redox potential of Cr(vi)/Cr(iii) in acidic conditions. This means that the reduction is thermodynamically favourable at lower pH values. To prevent the formation of by-products and the use of detrimental chemical reducing agents, eco-friendly alternative methods for Cr(vi) reduction have been developed. Among these methods, photocatalysis is regarded as one of the most promising, solar-energy-based solutions with low energy consumption, where the efficiency of Cr(vi) reduction depends primarily on the properties of the photocatalyst.^3^

Considering that, graphitic carbon nitride (g-C_3_N_4_, CN) has attracted significant attention owing to its activity under visible irradiation, high reduction potential of electrons in the conduction band, stability in acidic conditions, and non-toxicity.^4^ Furthermore, CN can be easily synthesized from nitrogen-rich precursors, making its use highly economical.^5^ Typically, CN is fabricated by the thermal condensation method using precursors with C–N core structure. Different chemical structures of the precursors result in divergent properties of the synthesized CN.^6^ For efficient photocatalysis, a photocatalyst with a high specific surface area is beneficial, as it provides more adsorption and reactive sites.^7^ It is acknowledged that CN obtained from urea exhibits the highest specific surface area and a surface enriched with reactive sites as a consequence of the formation of oxygen molecules during the thermal polymerization process.^8^ Nevertheless, just like any other single photocatalyst, CN has limited practical use mostly due to the high recombination rate of photogenerated charge carriers. In addition, a significant challenge is the inherently low dispersibility of CN, which leads to rapid agglomeration of its particles. This aggregation has been shown to reduce the available surface area, thereby hindering the efficient adsorption of target species for the removal from solution and, consequently, limiting its photocatalytic performance.

With this in mind, a variety of modification strategies have been employed, focusing on the combination with other materials or on morphology engineering and surface functionalization.^5,9^ While the doping with different elements can enhance the optical properties of CN and facilitate the utilization of visible light, the dopant states can concurrently function as recombination states for charge carriers.^10^ A promising approach to enhance the photocatalytic activity of CN involves the deposition of noble metals on the CN surface, leading to the formation of metal–semiconductor (MS) heterostructures. Metal nanoparticles (NPs) present on the CN surface exhibit a dual role: as both trapping and active sites. At the metal-CN interface, a Schottky barrier is formed, due to the disparity in work functions and Fermi levels of the two materials.^8,10,11^ The Schottky barrier enables the transfer of photogenerated electrons from the conduction band of CN to the Fermi level of the metal. This facilitates the separation and accumulation of electrons (e^−^) in the metal NPs, while the holes (h^+^) remain in the CN, preventing their recombination.^11^ Additionally, the formation of a metal–semiconductor heterostructure enables the utilization of metal NPs as a cocatalyst, thereby creating additional active sites. It is noteworthy that, while bulk CN can be activated by visible light, in the absence of a co-catalyst, the light absorption is markedly poor since it includes a small amount of visible light.^12^

Metal NPs exhibit a local surface plasmon resonance (SPR) effect, whereby irradiation with a wavelength much larger than their size induces an oscillation of conduction electrons in metals.^13^ It has been suggested that this effect is responsible for the enhancement of visible light absorption of CN. Although noble metal NPs have demonstrated excellent performance, their cost and scarcity have prompted research and development of alternative and more economical solutions. Among the various metals under consideration, Cu NPs emerged as a possible alternative, given their capacity to enhance the photocatalytic ability of the photocatalyst, functioning as a co-catalyst.^14^ It is known that Cu have higher work function than CN, meaning that an effective Schottky junction can be formed. However, the primary challenge associated with copper is its tendency to undergo surface oxidation in ambient conditions. Therefore, it is essential to regulate the synthesis process to produce Cu NPs in a reduced state.^8,15^ Fortunately, copper oxides (Cu_2_O and CuO) have also demonstrated particular promise owing to their narrow bandgap (*E*_g_ (CuO) = 1.2 eV and *E*_g_ (Cu_2_O) = 2.0 eV). Therefore, their presence on the CN surface could potentially enhance the photocatalytic efficiency owing to the formation of semiconductor(I)–semiconductor(II) heterojunction. Furthermore, under the illumination of visible light, the transformation of Cu-oxide by photogenerated electrons results in the formation of pristine Cu NPs, thereby ensuring the maintenance of the Cu surface in its metallic state under operational conditions.^15^ It has been demonstrated that the controlled generation of the semiconducting Cu_2_O layer over metallic Cu ensured the preservation of nearly unaltered SPR merits, provides protection of the Cu core from continuous oxidation, and contributes to additional co-catalytic function concerning the separation of charge carriers.

The photocatalytic performance of a specific metal–semiconductor heterostructure is influenced by the metal content, the size and distribution of the metal particles, and the interfacial interaction between the metal and the semiconductor. The interfacial interaction is primarily determined by the method of synthesis employed. A variety of methods have been utilized for the deposition of metal NPs on the photocatalyst surface, including chemical reduction, photodeposition, wet impregnation, solvothermal method, sputtering, and physical mixing.^11^ The most common method of synthesizing metal nanoparticles (NPs) on the surface of a photocatalyst is chemical reduction with NaBH_4_.^16^ However, achieving the desired size, shape, and uniform dispersion of the NPs requires precise control of the reduction process, which involves numerous steps.^17^ Photodeposition is also attractive because of its simplicity, using irradiation to activate the photocatalyst, followed by reduction of metal ions by excited electrons and deposition of NPs on the surface of the photocatalyst at room temperature without using harsh conditions.^10^ Notably, this method has demonstrated remarkable results, primarily in the context of noble metal deposition. However, transition metals, such as Cu, typically exhibit lower redox potential and electronegativity. Consequently, a higher overpotential is required for successful photodeposition. Therefore, the photodeposition process for Cu NPs is often challenging due to its low rate and efficiency. This results in incomplete reduction, thus requiring a substantially longer deposition time compared with noble metals.^11^

Among the various alternative methods, plasma processes have been demonstrated to be effective in synthesizing and immobilizing metal NPs, as well as in modifying the surface of CN.^17^ In particular, atmospheric pressure cold plasma (non-thermal plasma) is of special interest due to its efficacy, ease of control and modification, rapidity and environmental friendliness for the synthesis of various nanoparticles.^17^ Dielectric Barrier Discharge (DBD) plasmas are of notable interest due to their non-equilibrium state, which results in a high density of reactive species while maintaining a low temperature (<100 °C) throughout the system.^18,19^ The utilization of DBD plasma for the synthesis of metal NPs is founded on the reactive species it produces, including electrons, ions, and free radicals. These reactive species can act as reducing agents for metal precursors, thereby facilitating the reduction and deposition of metal NPs.^17,20^ On the other hand, cold plasma can also be utilized to oxidize metal ions to obtain metal oxide nanoparticles, particularly when aqueous solution is used.^21,22^ During the composite catalyst preparation by DBD plasma, a series of simultaneous processes occur, including the formation of defects, modifications of surface functional groups, and morphological changes. Concurrent with these processes is the homogeneous deposition of metal or metal oxide NPs, so the outcome is enhanced interaction between the composite components, leading to the formation of photocatalysts with significantly enhanced performance.^19^

Despite the use of noble metal NPs as CNs co-catalysts for the photocatalytic reduction of Cr(vi),^23–25^ the utilization of Cu-CN-based composites for this purpose has been very limited, although Cu-CN and bimetallic CN composites with Cu have been prepared using various methods and applied for hydrogen production and CO_2_ reduction.^26–28^ Cu NPs have been deposited on TiO_2_,^29^ but the use of Cu_2_O or CuO in the heterojunction with other semiconductors^30–33^ and CN^34^ is more common for the reduction of Cr(vi). Although the most frequent methods used for the deposition were chemical reduction, photodeposition, or precipitation in the case of copper oxides, different approach was applied due to the mentioned drawbacks in the case of copper NPs. The plasma method has already been recognized as a promising alternative to conventional methods for materials modification^21^ and NPs deposition,^35^ and in the case of copper and CN, it has been used to embed single Cu atoms in the CN structure.^36^

In this study, the properties and efficiency of photocatalytic Cr(vi) reduction of Cu-CN synthesized by the DBD plasma method were compared to those of the sample obtained by wet chemical reduction, as well as the bare and DBD plasma-treated CN. The plasma synthesis was selected because it introduces different functional groups and defects during the plasma modification process,^19^ which have the potential to enhance the stability of the heterojunction between Cu NPs and CN. The hypothesis is that DBD plasma will provide more homogeneous deposition and higher stability of Cu-based particles in comparison to the chemical reduction method.

## Experimental

### Materials and chemicals

Urea used for the CN synthesis was acquired from Thermo Fisher Scientific, Germany (99.5% pure) and citric acid monohydrate was purchased from Lachner, Czech Republic (99.8% pure). Copper chloride dihydrate, used as a Cu precursor, was purchased from Luphoma, while sodium borohydride was obtained from Acros Organics, Belgium. 1,5-Diphenylcarbazide was acquired from Sigma-Aldrich, USA (≥99% pure). Both deionized water (DI) and ethanol (≥99.5%) were used as the solvents throughout the work. Chemicals were used without any previous purification.

### Preparation of pristine CN

The pristine CN was prepared by the facile thermal polymerization method using urea as a precursor. In a typical synthesis, urea was placed in a covered alumina crucible and heated to 550 °C at a heating rate of 10°C min^−1^ and held for 4 h in air in a muffle furnace. Then the furnace was allowed to cool naturally to room temperature and the resulting solid sample was ground. The yellow powder obtained was collected and designated as CN.

### Preparation of Cu-CN by chemical reduction

The chemical reduction of Cu^2+^ ions in the presence of CN was performed by the following procedure: 2 g of the aforementioned CN was dispersed in 100 ml aqueous Cu^2+^ solution, prepared by dissolving CuCl_2_·2H_2_O (0.1 g Cu^2+^) in deionized water, and the solution was stirred for 1 h. The theoretical metal content was selected based on previous studies. In order to avoid excessive copper deposition while taking into account the fact that not all copper ions will be reduced and deposited, a concentration of 5 wt% was selected. Afterwards, 100 ml of NaBH_4_ solution was added, and the following reaction was assumed to have taken place: 2Cu^2+^ + BH_4−_ + 3H_2_O ↔ 2Cu + 2H_2_ + B(OH)_3_ + 3H^+^. The amount of NaBH_4_ used was twice the amount stoichiometrically required for the Cu^2+^ reduction, according to the reaction. The stirring was continued for another 30 min, after which the prepared suspension was centrifuged and washed with deionized water until the supernatant was clear. The product was dried overnight in the vacuum dryer at 60 °C. The resulting yellow powder was labelled as Cu-CN-cr.

### Preparation of Cu plasma decorated and plasma treated CN powders

Cu-decorated CN powders were prepared using a custom atmospheric plasma dielectric barrier discharge (AP-DBD) system. The experimental setup consisted of a vertically placed plasma torch with a coaxial geometry consisting of two concentric hollow quartz tubes. The plasma was generated between a Pt-coated inner tube and an Al-foil-coated outer tube by injecting 10 slm of argon and applying a 50 kHz, 20 W sinusoidal voltage to the outer electrode, with the inner tube grounded.

CuCl_2_·2H_2_O was dissolved in ethanol/water mixture (50/50, 0.5 g l^−1^). This solution was injected at a rate of 0.1 ml min^−1^ using a syringe pump system. Microdroplets were generated using an ultrasonic nebulizer (Sono-Tek®, 1 W, *f* = 120 kHz). The aerosol was then delivered into the plasma post-discharge using Ar (10 slm), where the microdroplets reacted with energetic plasma species.

Pure CN powder was dispersed in water (1.75 mg CN in 40 ml H_2_O). It was then exposed to the plasma at a distance of 5 cm and stirred continuously to ensure optimal CN decoration. The setup was optimized to ensure the best result in terms of uniformity of dispersion and size distribution of NPs. This method has been successfully applied to the synthesis of Au [20], FeO_*x*_ [22], as well as the decoration of P25 TiO_2_ with Au NPs [35]. This sample is referred to as CN-Cu-pl in the article. A similar process was also performed without Cu precursor injection to evaluate the influence of plasma only on the CN powders. This powder is referred to as CN-pl.

### Characterization techniques

The surface morphology of the samples was observed with field emission scanning electron microscopy, FESEM, using Tescan Mira3 XMU at a working voltage of 20 kV. Before analysis, samples were sputter-coated with gold to ensure their conductivity. Transmission electron microscopy (TEM) analysis was performed on the FEI Talos F200X microscope (Thermo Fisher Scientific, Waltham, MA, United States) at an accelerating voltage of 200 kV. Additionally, selected area electron diffraction (SAED) was performed. The observation of the sample's morphology was conducted through the dispersion of a small amount of the powder in ethanol. A drop of the resulting dispersion was then applied onto a grid and left to dry overnight. For image investigation and calculations of *d*-spacing values ImageJ software was used. Fourier transmission infrared spectrometer (FTIR) was used for the characterization of the surface functional groups of the obtained samples, using Thermo Scientific Nicolet iS10 in attenuated total reflection mode and in the wave number range of 400–4000 cm^−1^. Raman spectra were recorded using DXR Raman Microscope (Thermo Fisher, USA) at room temperature, with an excitation of 780 nm laser light. X-ray photoelectron spectroscopy (XPS) was used for analysing the surface elemental composition of the samples with Kratos Axis Ultra DLD X-ray photoelectron spectrometer with a monochromatized Al Kα line source (150 W). The binding energies were referenced to the adventitious carbon at 285.0 eV. EPR spectra were recorded at room temperature using a Bruker ELEXSYS-II E540 spectrometer (Bruker, Rheinstetten, Germany) operating in the X-band range, with the following parameters: magnetic field center at 3513 G, microwave power 10 mW, microwave frequency 9.85 GHz, modulation frequency 100 kHz, and modulation amplitude 2 G. Data analysis was performed using Xepr software, version 2.6b.84 (Bruker BioSpin, Rheinstetten, Germany). The optical properties of the samples were studied using UV-Vis diffused reflectance spectroscopy (DRS) and the spectra was recorder on a Shimadzu 2600 UV-Vis spectrophotometer with integrated ISR-2600-Plus sphere in the wavelength range of 200–800 nm and with BaSO_4_ as the standard reference. To calculate absorption spectra from the reflectance data, the Kubelka–Munk function given as *F*(*R*) was used. Photoluminescence (PL) spectra were recorded using a Fluorolog-3 FL3–221 (Horiba Jobin-Yvon) spectrofluorometer system with a 450 W Xenon lamp as the excitation source and an excitation wavelength of 370 nm.

Photoelectrochemical measurements were carried out using screen-printed carbon electrodes (SPCEs, Metrohm DropSens DRP-11L) connected to an electrochemical workstation (Metrohm, µStat 400 Bipotentiostat/Galvanostat). The SPCEs consisted of an integrated Ag/AgCl reference electrode, a carbon working electrode (geometric diameter of 4 mm), and a carbon counter electrode. For the preparation of catalyst inks, synthesized samples were dispersed in a mixed solvent containing 320 µL of isopropyl alcohol, 80 µL of deionized water, and 20 µL of 5 wt% Nafion® solution, followed by ultrasonication. Subsequently, 5 µL of ink was drop-cast onto the surface of the working electrode and dried at room temperature, resulting in a catalyst loading of 2 mg cm^−2^. Transient photocurrent (TPC) measurements were recorded by chronoamperometry under chopped illumination at −0.4 V *vs.* Ag/AgCl with 20 s light on/off cycles in 0.5 M Na_2_SO_4_ electrolyte, using an Osram Vitalux lamp (300 W) as the light source and 400 nm UV cut off filter. Cyclic voltammetry (CV) measurements were performed in the potential range from −1.5 to +1.5 V *vs.* Ag/AgCl at a scan rate of 50 mV s^−1^.

The photocatalytic performance of bulk CN, Cu-CN-cr, CN-pl and Cu-CN-pl photocatalysts towards Cr(vi) reduction under visible irradiation was investigated as follows: the 5 mg l^−1^ Cr(vi) solution of pH = 3 was prepared by dissolving K_2_Cr_2_O7 (Hemo, Belgrade (99.9%)) in deionized water and using concentrated (98%) H_2_SO_4_ (Lachema, Czech Republic (98%)) for pH adjustment. In detail, 40 ml of Cr(vi) solution and 0.07 ml of citric acid solution (20 g l^−1^) (Lachner, Czech Republic (99.8%)), used as a hole scavenger, were poured into the reactor containing 0.01 g of photocatalyst. This reactor, used for photocatalytic experiments at ambient temperature, had an outer jacket for circulating cooling water to avoid overheating. The suspension was stirred continuously throughout the experiments, while before irradiation it was stirred in the dark for 30 min to reach adsorption–desorption equilibrium. The simulation of visible light was achieved by utilizing a high-pressure Mercury Reprographic lamp (Philips, 125 W) with a filter (GG400 Farbglass SCHOTT) cutting off the light below 400 nm. The lamp was placed above the reactor at a constant distance and intensity during the experiments. After every 15 min of irradiation, 1 ml of the suspension was taken out and filtered to remove the sample. The Cr(vi) concentration of the filtered solution was measured using a UV-Vis spectrophotometer (Shimadzu UV-1800) with a characteristic absorption peak at 542 nm and 1,5-diphenylcarbazide (Sigma-Aldrich, USA (≥99%)) as an indicator.

## Results and discussion

### Characterization techniques

**Fig. 1:**
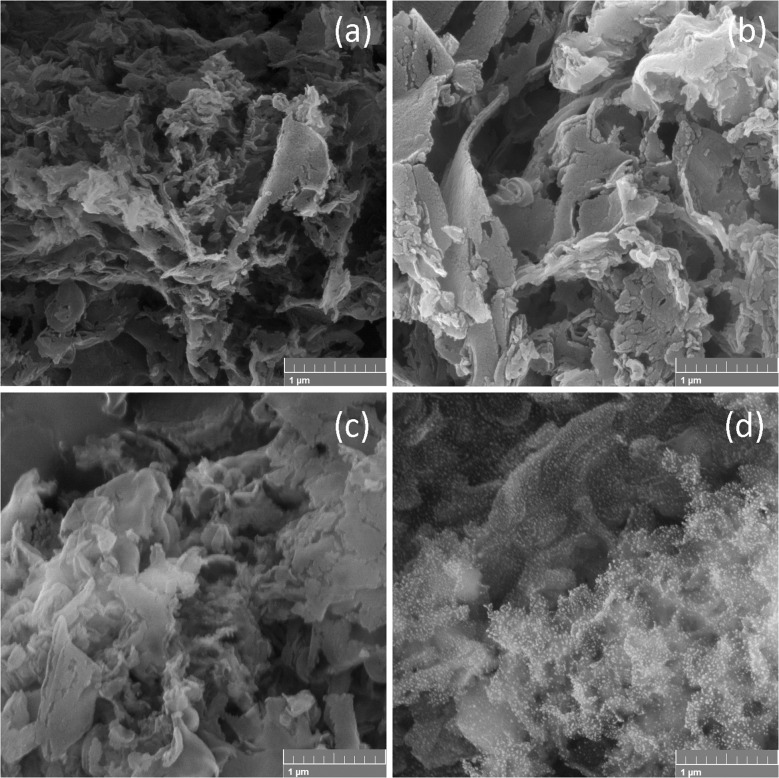
FESEM images of the (a) pristine CN (b) Cu-CN-cr (c) CN-pl and (d) Cu-CN-pl.

The morphologies of the synthesised samples are presented in Fig. 1. Pure CN shows a graphite-like layered structure composed of curled and agglomerated particles with wrinkled surfaces. As shown in the figure, all the CN-based photocatalysts showed the same layered structure. The surface of the Cu-CN-cr sample seems slightly rougher than of CN, although the deposited nanoparticles could not be seen. A comparison of CN-pl and CN reveals no observable differences, suggesting that the DBD plasma discharge does not induce alterations in the CN powders, probably due to the mild conditions of the plasma treatment. On contrary to this, when DBD plasma was used in the synthesis of Cu-CN-pl, the surface of CN layered particles was mostly uniformly covered with nanoparticles of about 20 nm in size. Since copper can be present in different oxidation states, it cannot be accurately claimed that these are Cu nanoparticles, and not CuO_*x*_. Nevertheless, it is clear that the use of plasma directly on the Cu precursor solution provided homogeneous deposition of very fine non-agglomerated particles on the CN surface. As it has been suggested that plasma treatment can be used for surface treatment and functionalization of materials, it is possible that this provided a plenty of sites for the NPs attachment, causing very good dispersion of NPs.^19^

**Fig. 2:**
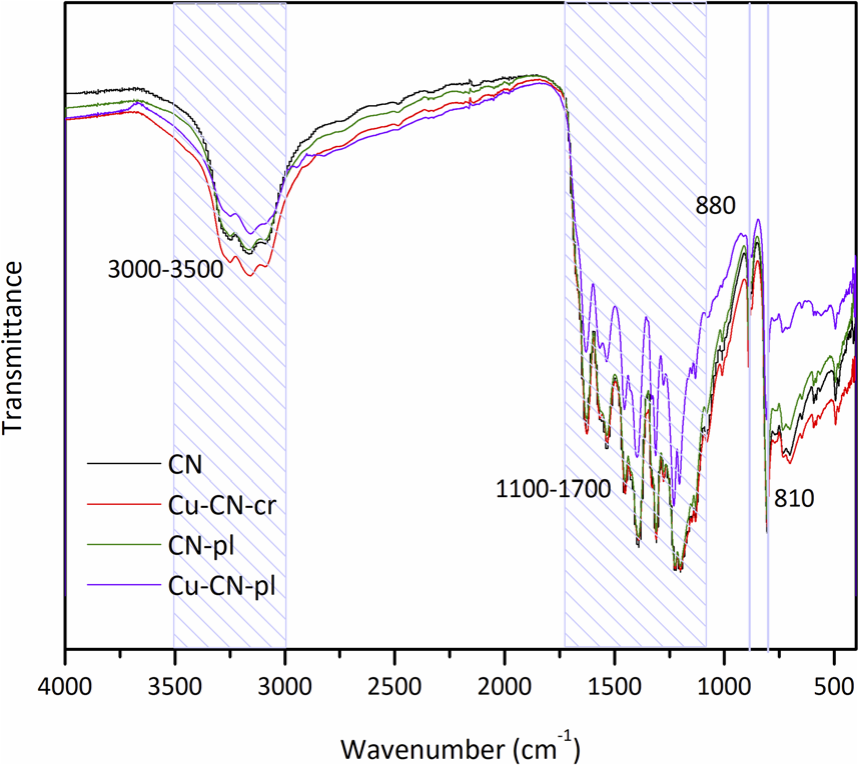
FTIR spectra of the CNs.

FTIR spectroscopy was utilized to investigate the surface properties and to identify the functional groups of the synthesized samples, including any potential modifications that occurred after plasma treatment and/or Cu deposition (Fig. 2). The broad band observed in all samples within the range of 3000–3500 cm^−1^ originates from N–H stretching vibrations in free amino groups, but also from O–H groups in adsorbed water molecules on the surface.^37,38^ The presence of characteristic triazine units is indicated by a series of bands present from 1100 to 1700 cm^−1^, attributed to the C=N and C–N aromatic stretching vibrations.^16,37^ The intense peak at 810 cm^−1^, which is also observed in all the FTIR spectra, can be assigned to the out-of-plane bending vibration of the tri-s-triazine ring system,^16,39^ while the smaller sharp peak at 880 cm^−1^ is attributed to the N–H rocking vibration from the incomplete condensation of amino groups.^37,40^ The present results indicate that all the CN-based samples are composed of triazine units, and that the basic structure of CN was maintained after modifications. The spectra of the sample Cu-CN-cr show no significant difference compared to the pure CN, meaning that this modification did not lead to the formation of the new bands. However, the absence of bands related to the Cu–O bond in the 400–800 cm^−1^ range, which could be present due to oxidation of Cu particles, could be attributed to the relatively low Cu content. An alternative explanation is that, rather than forming Cu NPs, Cu atoms/ions were incorporated into the CN structure, which is in agreement with the results of the FESEM analysis. Furthermore, the plasma-treated CN (CN-pl) exhibited analogous spectra to the CN, thereby suggesting that the chemical bonds on the surface of the CN remained unaltered by the plasma treatment. Another possibility is that recently formed surface functional groups reoriented themselves to settle for a lower state of energy on the plasma-treated surface of the CN, which is known as the aging effect.^41^ It has been observed that, over time and when exposed to air, carbon materials undergo a gradual loss of surface activity. This phenomenon underlies the challenges associated with maintaining the generated functional groups on the surface, and the time interval between synthesis and FTIR analysis is likely to be a contributing factor. Despite the absence of any new peaks in the spectrum of the Cu-CN-pl sample, a decrease in intensity was observed in the bands within the 3000–3500 cm^−1^ range, which may be a consequence of the attachment of Cu. Basically, the obtained FTIR spectra for both Cu modified samples showed no signs of any disruption upon the Cu introduction meaning that no new Cu-based groups were formed. However, in the case of Cu-CN-pl, the bands between 1100–1700 cm^−1^ appear to be modified, as their intensity is altered with respect to the pure CN.^42^ This further suggests the interactions of Cu with CN heterocycles and possible breaking of the aromatic C–N structures.^38^

To further study the vibrational properties of CN samples, Raman spectroscopy was used. The Raman spectra of the samples, obtained by using a 532 nm laser as the excitation source, is shown in Fig. S1. It is important to point out that the application of visible Raman spectroscopy for CN is accompanied by significant fluorescence, which is responsible for obtaining unsatisfactory resolution, especially in region 1200–1700 cm^−1^. However, in the case of Cu-CN-pl sample, it can be noticed that fluorescence is less intense. It is known that during the recombination of photogenerated e^−^ and h^+^, light is being emitted, meaning that this sample could have lower recombination rate of the charge carriers.^43,44^ The intense peaks at 707 cm^−1^ and 757 cm^−1^ are attributed to the heptazine ring breathing modes,^45,46^ while the peak at 1233 cm^−1^ is related to the typical stretching vibration modes of C=N and C–N heterocycles.^45^ These peaks are present in all samples, though they are less intense after modification with Cu. This indicates that the basic CN structure was retained in the modified samples, as shown by the FTIR spectra (Fig. 2), though with some changes. The decrease in peak intensity suggests that defects were introduced, despite the absence of peaks related to Cu species. Still, the D band (∼1350 cm^−1^) and G band (∼1600 cm^−1^) which are assigned to the disordered sp^2^ C and in-plane vibration of graphitic layers, respectively, couldn't be noticed due to the strong fluorescent background.^47^

**Fig. 3:**
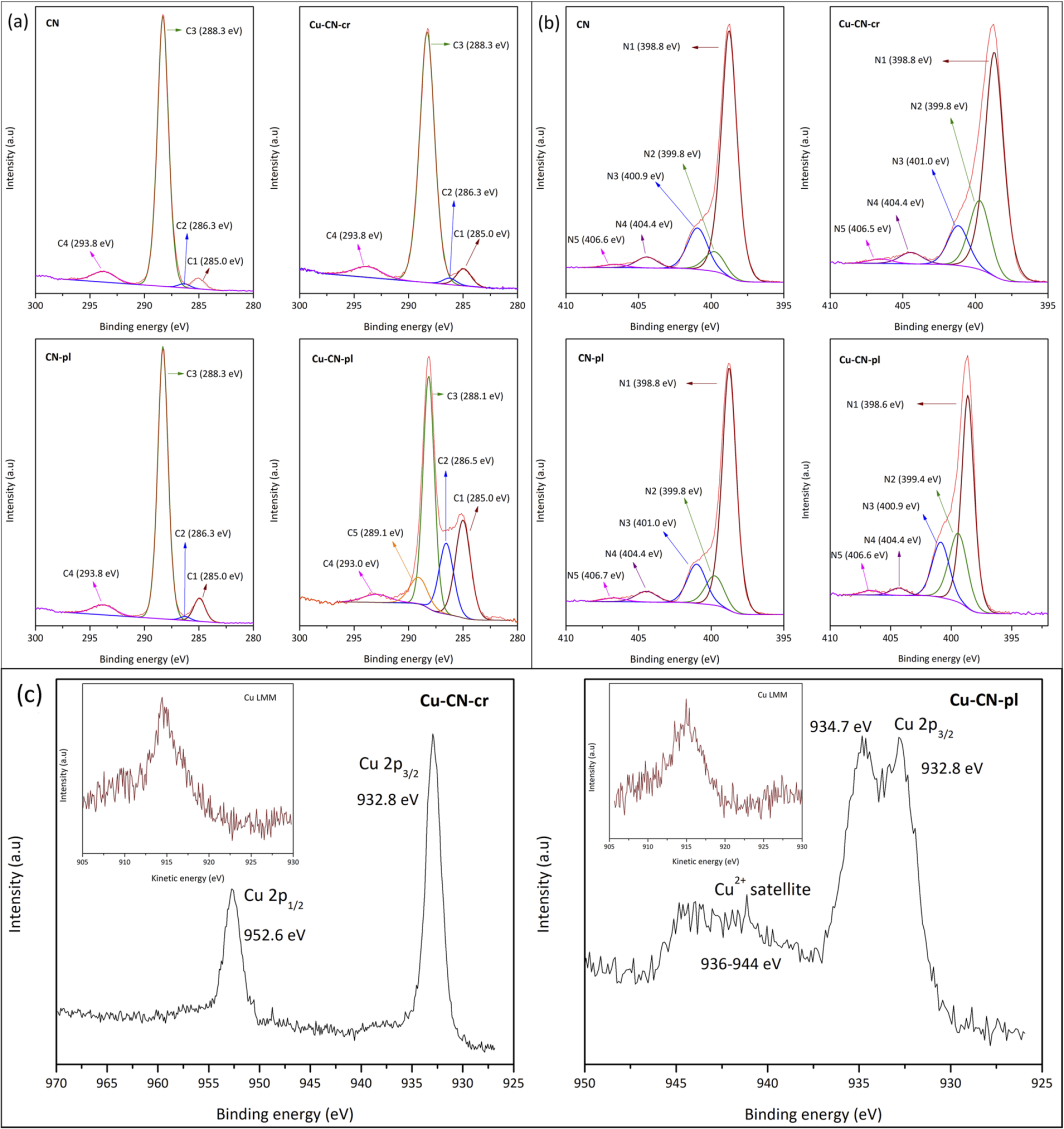
High resolution XPS spectrums of (a) C 1s and (b) N 1s for CN, Cu-CN-cr, CN-pl and Cu-CN-pl; (c) High resolution Cu 2p XPS spectrum and Cu LMM spectrum (the inset) of Cu-CN-cr and Cu-CN-p.

X-ray photoelectron spectroscopy (XPS) was used to analyse the elemental composition and chemical states of the pure CN and modified samples (Fig. 3). The elements C, N and O were found in all samples, while Cu was detected in the Cu-CN-cr and Cu-CN-pl samples. The high resolution XPS spectra of C 1s region of the CN (Fig. 3a) can be deconvoluted into four peaks at 285.0, 286.3, 288.3 and 293.8 eV. The main peak at 288.3 eV (C3) is assign to sp^2^-bonded carbon in the N=C–(N)2 group in heterocycles, which is the major carbon specie in the CN (88.3% according to the peak area).^48^ The peak at 285.0 eV (C1) is assigned to the surface adventitious carbon species (C=C/C–C) (3.4%), while the weak peak at 286.3 eV (C2) corresponds to the C–O/C–O–H bonds (1.3%) from adsorbed CO_2_ or due to some substitution of nitrogen with oxygen during the CN synthesis.^40^ The peak at 293.8 eV (7.0%) is assigned to the π–π* satellite bonds (C4).^38,49,50^ The N 1s spectra of CN is deconvoluted into five peaks (Fig. 3b) with binding energies of 398.8 (N1), 399.8 (N2), 400.9 (N3), 404.4 (N4) and 406.6 (N5) eV.^51^ The main peaks N1 (72.6%) and N3 (15.0%) are characteristic of sp^2^-hybridized N involved in the triazine rings (C=N–C) and amino groups (C–N–H), respectively. The N2 peak (7.2%) is attributed to tertiary N bonded to carbon atoms in the form of N-(C)3. The peak at 404.4 eV can be assigned to NO_*x*_ functionalities due to contamination, and peak at 406.6 eV to π–π* satellite bonds.^52^

For the Cu-CN-cr and Cu-CN-pl samples, it is important to determine the oxidation state of the present Cu (Fig. 3c). In the case of Cu-CN-cr, the two asymmetric peaks of the Cu 2p spectra located at 932.8 and 952.6 eV are ascribed to Cu 2p_3/2_ and Cu 2p_1/2_ peaks of Cu^+^ or metallic Cu.^38^ In this sample there is no satellite structure characteristic of the Cu^2+^ state, so its presence can be excluded.^14^ To determine whether Cu^+^ or Cu is present, the Cu LMM spectrum was examined. The results showed that the spectrum (Fig. 3c (the inset)) consists of the broad band between 910 and 920 eV, with a Cu LMM peak at K. E. ∼914.7 eV, characteristic of Cu^+^/Cu_2_O, which is the result of the reduction of Cu^2+^ from CuCl_2_ with NaBH_4_. Taking into account the FESEM micrograph of the sample (Fig. 1b), where the NPs cannot be seen, the incorporation of Cu^+^ ions in the CN structure, most likely chemically coordinated to the N atoms, can be assumed.^53^ In the case of Cu-CN-pl, the Cu 2p XPS spectra present similar peaks, but the Cu 2p_3/2_ peak shows a second component at 934.7 eV, as well as the broad shake-up satellite peak at 936–944 eV, which are related to the presence of Cu^2+38^. According to literature, this second peak can be undoubtedly ascribed to Cu(OH)_2_.^54,55^ Thus, copper is present as a mixture of Cu(OH)_2_ (934.7 eV) and Cu_2_O (932.8 eV). Based on these results, it is likely that CN surface is covered with Cu_2_O/Cu(OH)_2_ NPs, considering also FESEM micrograph (Fig. 1d). It is possible that the plasma conditions were too mild to reduce Cu^2+^, or more likely that Cu^+^ or Cu(0) was oxidized with highly oxidative species that are formed in atmospheric plasma, such as O_3_, OH˙, H_2_O_2_, which can react in both the gas and liquid phases.^56^ The presence of Cu(OH)_2_ component is thus very likely due to the water used as solvent for the sprayed solution and the medium of CN suspension during the decoration of particles for the Cu-CN-pl sample. Considering that the synthesis was performed in aqueous-ethanol media, the presence of Cu oxides/hydroxides was rather expected.^21^

Regarding the C 1s region of the Cu-CN-cr sample, the positions and intensities of the present peaks were not affected much by the procedure of Cu^2+^ reduction with NaBH_4_ and presumed incorporation of Cu^+^ in the structure, but it just slightly increased the content of carbon from C–O component (from 1.3 to 1.9%). It is similar for N 1s, where the N1 peak was a little more intense (74.0%), while N3 peak was a little less intense (12.9%). These findings indicate that the incorporation of Cu^+^ ions practically did not affect the structure of CN, suggesting that no C or N substitution occurred. Taking into consideration the electroneutrality of the structure, it can be assumed that Cu^+^ ions have substituted H^+^ ions in the amino groups.

Comparing the samples obtained using plasma, CN-pl and Cu-CN-pl, significant differences are observed, primarily for C 1s. After just plasma treatment of CN, the peaks in the C 1s region retained their positions, but the intensity of the C1 peak (C=C/C–C) is quite increased (7.8%) and the intensity of C3 (N=C–(N)2) is proportionally reduced (84.2%). On the other hand, in the case of the Cu-CN-pl sample, the applied modification led to a certain negative shift in the position of C3 (∼0.2 eV) peak and to significant changes in the peak's intensity, in comparison to both CN and CN-pl. It is known that the shifts in binding energy, both positive and negative, reflect changes in electron density-decreasing in the first case and increasing in the second-highlighting the different electron transfer pathways in the samples. In the case of the Cu-CN-pl sample, this means that the electron density around the C atoms in the CN framework is enhanced in comparison to CN and CN-pl, indicating some changes in the vicinity of the C atom. Also, the intensity of the peaks C1 (C=C/C–C) and C2 (C–O/C–O–H) increased drastically, while the intensity of the peak C3 (N=C–(N)2) species decreased, meaning that the part of the C–N bonds was replaced by C–C or C–O bonds. The C1 peak might also be stronger because of the carbon that forms around the Cu-based NPs as a result of using the ethanol-based precursor solution.^20,22,35^ In addition, the peak at 289.1 eV is observed, which is assigned to the N-CO_*x*_ or N=C-(NO), probably due to the oxidation of the N=C-(N)2 component.

Similar as for C 1s, N 1s region for sample CN-pl was not changed much in comparison to CN: the position of all the peaks was maintained, but the intensity of the peak N1 (C=N–C) was slightly decreased and the peak N2 (N–C3) was increased. This, the deconvolution of both C 1s and N 1s indicates a small decrease in the proportion of C=N–C bonds in the CN-pl, meaning that the plasma treatment led to some disruption of the aromatic rings in the CN structure. Much larger changes in this region were observed for the Cu-CN-pl sample, as for C 1s region, where the peaks also showed a negative shift with respect to pure CN and their intensity was also affected. As same as for the CN-pl sample, the intensity of the N1 (C=N–C) peak was decreased, but to a much greater extent, while the intensity of the peak N2 (N-(C)3) increased. This indicates that the content of sp^2^-bonded nitrogen significantly reduced, probably because it has the weakest bonding and stability,^57^ while the content of sp^3^-bonded nitrogen increased. It should be noted that the content of nitrogen-oxygen groups is very low, in contrast to the content of carbon–oxygen groups, meaning that the oxygen introduced by plasma treatment is mainly connected to carbon in the structure, forming oxygen-containing functional groups, like C–OH. On the other hand, plasma treatment introduced nitrogen defects, mainly concentrated at the sites with sp^2^-bonded nitrogen.^58^ These functional groups and vacancies are undoubtedly the binding sites for copper ions, from which Cu_2_O/Cu(OH)_2_ NPs grow over time. The registered negative shift of the C 1s and N 1s peaks is due to bond breaking and binding of ions/NPs.

The question is why the plasma treatment without Cu did not cause structural changes of such intensity as in the sample with Cu. Previous studies have shown that the functional groups and defects generated on plasma-activated surfaces have a short shelf life, as they tend to reorient themselves to attain lower energy states.^41^ In the presence of Cu species, these functional groups and defects are stabilized owing to the formation of the bonds with these species, but in the absence of Cu-species, as for the sample CN-pl, these activated sites have been healed over time.

The elemental composition of the samples was determined from the narrow scan, and the values given in Table 1 are the averages of the two determinations. The obtained C/N atomic ratio of 0.77 for pure CN is very close to the ideal stoichiometric (0.75).^40^ The other samples showed higher ratios, especially after plasma treatment. The Cu-CN-pl sample showed a drastically lower N content and slightly higher C content compared to pure CN, resulting in the highest C/N ratio (1.64), confirming the formation of nitrogen vacancies and deposition of additional carbon with Cu-based NPs. The oxygen content was higher in all the modified samples, but the greatest increase was observed in the Cu-CN-pl sample, which is in accordance with the previous discussion. The formation of Cu_2_O and Cu(OH)_2_ also contributes to the increase in oxygen content. Some part of the O is from the SiO_2_, as Si is also detected from the wafer used as a holder during synthesis. The Cu content is roughly similar in both the Cu-CN-cr and Cu-CN-pl samples, thus, potential differences in photocatalytic efficiency can be attributed to differences in the type rather than the amount of Cu.

**Table 1:** Elemental composition (at%) obtained from narrow scans, averaged over two measurements

Sample	C	Cu	N	O	Cl	Si	C/N
CN	43.2	—	56.2	0.6	—	—	0.77
CN-pl	45.2	—	53.3	1.5	—	—	0.85
Cu-CN-cr	43.1	1.2	54.4	1.3	—	—	0.79
Cu-CN-pl	45.5	0.8	27.8	18.5	1.2	5.8	1.64

**Fig. 4:**
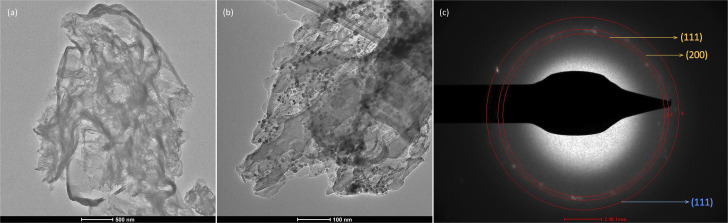
TEM images of (a) Cu-CN-cr, (b) Cu-CN-pl and (c) SAED pattern at the Cu-CN-pl interface.

The structural features of samples containing Cu were further analyzed by TEM (Fig. 4). The image of Cu-CN-cr (Fig. 4a) clearly shows that no NPs were deposited on the CN surface. This finding is consistent with the FESEM and XPS results. Meanwhile, the image in Fig. 4b confirms the successful formation of Cu-based nanoparticles in the Cu-CN-pl sample. These nanoparticles are randomly dispersed on the CN surface and range in size from 10 to 20 nm. It is possible that smaller NPs were not visible in the FESEM image (Fig. 1d) because they were wrapped in CN nanosheets and/or obscured by larger NPs. SAED analysis revealed that the deposited NPs were polycrystalline (Fig. 4c). The interplanar spacing (*d*) was calculated from the SAED image (Fig. 2d) and is equal to 0.240 nm, 0.211 nm, and 0.202 nm. These values correspond to the (111) and (200) planes of Cu_2_O (yellow colour in Fig. 4d), and the (111) plane of metallic Cu (blue colour in Fig. 4d), respectively. Due to the proximity of the *d*-values of Cu_2_O (0.240 nm for (111)) and Cu(OH)_2_ (0.230 nm for (022) and 0.250 nm for (111)), it is difficult to determine the presence of either species. However, the observed (200) plane signal suggests the presence of Cu_2_O NPs.^58,59^ It is possible that Cu(OH)_2_ is present as an amorphous phase on the surface of the NPs. Considering the XPS results, it is clear that Cu is present in different oxidation states. Nevertheless, the TEM results also imply the presence of metallic Cu. The Cu NPs may be part of a core/shell structure where the outer layers are oxidized, thereby preventing surface-sensitive XPS analysis from detecting metallic Cu in the particle core.

In order to obtain more information about the electronic structural features of CN and CN-based nanomaterials, X-band EPR spectroscopy was used. Room temperature EPR spectra (Fig. S2) measured in darkness, revealed that all samples showed a single Lorentzian line centering at *g* = 2.0034. Therefore, it can be concluded that only one type of paramagnetic species is present, namely unpaired electrons on π-conjugated CN aromatic rings.^60,61^ In addition to the EPR signal that was recorded in the dark, all the samples were exposed to simulated solar light for a period of 60 s. Also, the relative intensities of the EPR signals were summarized in Table S1. The heightened intensity of the EPR signal signifies the generation of novel paramagnetic species subsequent to the illumination, which are presumably photogenerated electrons.^60^ Following the plasma treatment, the CN-pl sample exhibited enhanced signal intensity compared to the untreated CN, a phenomenon attributed to the introduction of defects. The results of the XPS analysis indicated a decrease in nitrogen content, thereby confirming the loss of nitrogen-containing species and the subsequent formation of nitrogen-vacancies. This process ultimately leads to the generation of unpaired electrons,^62,63^ and higher signal intensity. On the contrary, after introducing Cu into the Cu-CN-cr sample, the intensity of the EPR signal decreased, and no additional signal could be observed. This finding confirms the presence of Cu^+^, as Cu^+^ does not have unpaired electrons (it has a 3d^10^ configuration) and is EPR silent. Additionally, the reduced EPR signal intensity from CN after the introduction of Cu^+^ could be the result of electron transfer from the CN π-conjugated system to the Cu^+^ species. In the case of the Cu-CN-pl sample, the EPR signal intensity was significantly higher than that of the other samples. Therefore, its spectrum is not included in Fig. S2, and the corresponding intensity is not listed in Table S1. It is known that Cu^2+^ has a strong EPR signal due to its 3d^9^ configuration and unpaired electrons.^64^ Hence, it is likely that Cu^2+^ is present in Cu-CN-pl sample, which was already indicated by XPS analysis.

**Fig. 5:**
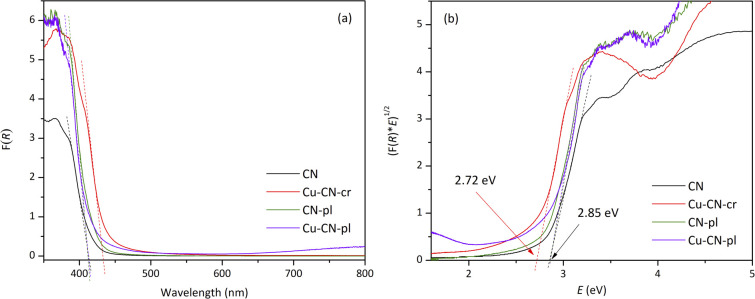
(a) UV-Vis absorption spectra of the CNs and (b) the corresponding Tauc plots.

To analyse the optical properties of the samples, UV-Vis diffuse reflectance spectra was obtained (Fig. 5a). Based on the Kubelka–Munk equation^65^ and the resulted Tauc plots, the optical band gap (*E*_g_) values were estimated (Fig. 5b). The obtained band gap energy for pure CN was 2.85 eV. This value is larger than typical values reported for CN (∼2.7 eV), but it was also shown that the *E*_g_ values of urea-derived CNs are significantly larger than of CNs derived from other precursors.^66–68^ After plasma treatment (CN-pl), the absorption edge remained the same. It has been reported that plasma modification of CN can cause the formation of nitrogen vacancies responsible for narrowing the band gap, but this wasn't the case here, obviously due to very small changes of the structure of CN-pl in comparison to CN, as it was shown by XPS analysis. It can be noted that the Cu-CN-cr sample shows a slight red shift so that the band gap energy is about 2.72 eV, indicating that Cu^+^ incorporation in the CN structure, which was shown by XPS, affects the electronic structure, leading to absorption in the longer wavelength regions in comparison to CN. The absence of SPR band in the visible region, characteristic for Cu(0) nanoparticles,^11,69^ is one more confirmation that Cu NPs were not formed during chemical reduction. Despite the presence of deposited Cu-based NPs in the sample Cu-CN-pl and significant changes in the CN structure, as evidenced by prior analyses, DRS spectrum of this sample is comparable to that of CN-pl and *E*_g_ values are the same. Though, there is a small enhancement of the light harvesting ability for Cu-CN-pl in the near-infrared region (700–800 nm), which can be related to the SPR effect of Cu NPs and defect sites associated with the loading of Cu NP.^14,26^ This suggests the presence of metallic Cu, probably in the core of NPs, as suggested by TEM analysis.

**Fig. 6:**
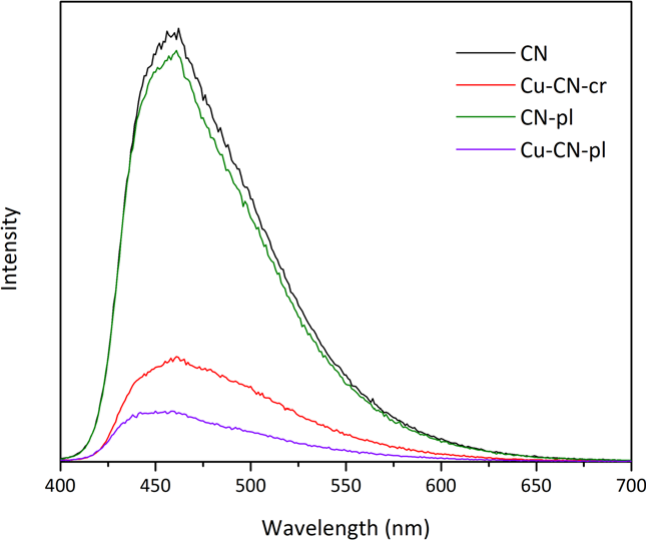
PL emission spectra of the CNs.

PL spectroscopy is considered one of the most convenient techniques for investigating the recombination rate of electrons and holes. Based on the obtained spectra (Fig. 6), it can be seen that pure CN and CN-pl have similar PL peak intensities, which is expected, considering the previously acquired characterization results for these samples. However, both of the samples with Cu showed lower PL peak intensities, meaning the lower recombination rate of charge carriers, especially for the Cu-CN-pl sample. Clearly, the deposition method had a significant influence on the separation efficiency of the e^−^ and h^+^, since Cu-CN-pl sample had quite lower PL peak intensity compared to Cu-CN-cr sample.

**Fig. 7:**
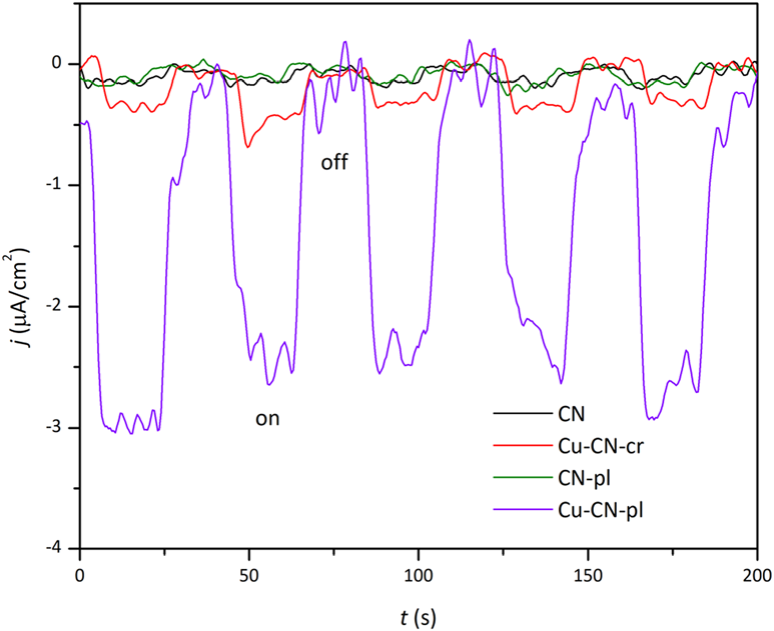
Transient photocurrent (TPC) response (*j*–*t* curves) of the synthesized samples under chopped light irradiation, measured at a potential of −0.4 V *vs.* Ag/AgCl in 0.5 M Na_2_SO_4_ solution.

To give further explanation for photo-sensitivity and transport efficiency of charge carriers, TPC measurements were performed (Fig. 7). The obtained results indicate distinct differences in the TPC response of the samples, following the order: Cu-CN-pl ≫ Cu-CN-cr > CN ≈ CN-pl. As can be seen, samples with copper exhibit significantly higher photocurrent densities, compared to CN and CN-pl, with the biggest difference for the Cu-CN-pl sample. This can be attributed to the more efficient separation of photoinduced electron–hole pairs and the suppression of their recombination, which is consistent with the given PL spectra (Fig. 6). Based on the DRS and PL results, both of the samples with Cu have similar *E*_g_ values and reduced PL peak intensities, but Cu-CN-pl sample showed a significantly higher photocurrent response compared to Cu-CN-cr.

### The efficiency of the CN-samples in the photocatalytic Cr(vi) reduction

**Fig. 8:**
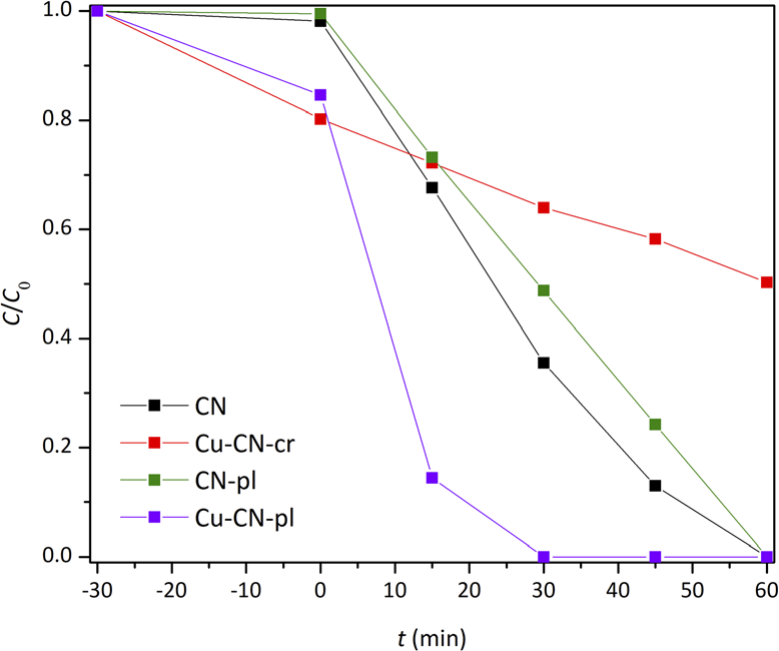
Photocatalytic reduction of Cr(vi) under the visible irradiation with the CNs.

The photocatalytic efficiency of pure CN and CN-based samples was evaluated through the photocatalytic reduction of Cr(vi) in an acidic environment and under visible irradiation (Fig. 8). Typically, Cr(vi) reduction is more effective at lower pH levels, but an extremely low pH can create harsh conditions. Therefore, a pH of 3 was selected to balance efficiency and conditions. In all the experiments, citric acid was used as a hole scavenger. Prior to irradiation, an adsorption–desorption experiment was conducted, with the solution maintained in the dark for 30 min to ensure equilibrium between the photocatalyst and Cr(vi) species. It was observed that the decrease in the Cr(vi) concentration in the dark for CN and CN-pl was negligible, while both Cu-CN-cr and Cu-CN-pl exhibited a more pronounced decrease. This phenomenon could be attributed to the enhanced adsorption capacity of these samples for Cr(vi), or alternatively, to the occurrence of chemical reduction of Cr(vi) by Cu-species present on the CN surface. In the case of Cu-CN-cr, it is possible that Cr(vi) is reduced by Cu^+^ ions present in the structure, as shown by XPS analysis. On the other hand, it is likely that Cr(vi) is adsorbed on the surface of CuO_*x*_ particles in the sample Cu-CN-pl, which probably has Cu(OH)_2_ on the surface, while Cu_2_O is present beneath the surface.

The role of electrons in the photocatalytic reduction of Cr(vi) was investigated using AgNO_3_ as an electron scavenger for CN, as an example (Fig. S3). The photocatalytic activity was almost completely inhibited, indicating that electrons are the primary active species involved in the reaction. The photocatalytic reduction of Cr(vi) followed first-order kinetics (Fig. S4) and the rate constants (*k*) were determined by the slope of the fitted line. Plasma-treated CN (CN-pl) showed activity comparable to that of pure CN, which was somewhat expected based on the nearly identical characterization results. A slight deterioration of the photocatalytic efficiency of CN-pl can be attributed to a small decrease of the content of sp^2^-bonded nitrogen, as shown by XPS, *i.e.* to the partial breaking of aromatic C–N structures and a consequent decrease of π delocalization.^70^ The sample Cu-CN-cr showed not only the highest decrease in the Cr(vi) concentration during the experiment in the dark, but also the lowest photocatalytic activity under simulated visible light, regardless of the improved visible light absorption (Fig. 5), confirming that the light absorption capacity of the photocatalyst is not the only critical factor affecting its photocatalytic performance.^71^ In order to further examine why the photocatalytic activity of Cu-CN-cr sample was so reduced, despite the predisposition for improved activity, cyclic voltammetry (CV) was employed as a qualitative indicator of sample stability before and after TPC measurements (Fig. S5). Significant differences were observed in the CV curves of the Cu-CN-cr sample before and after TPC measurements (Fig. S5a), indicating its low photostability. Having in mind very low photocatalytic efficiency of this sample, it is possible that most of the photogenerated electrons were spent for the reduction of Cu^+^ in the structure, not for the Cr(vi) reduction from the solution.

On the contrary, the Cu-CN-pl sample showed exceptional photocatalytic efficiency under the same conditions as for other samples. Compared with the Cu-CN-cr, the amount of Cu was similar but the photocatalytic reduction much more effective. Interestingly, DRS analysis did not show any change in the band gap energy, nor the presence of another absorption edge that would suggest the formation of heterojunction, but just a small enhancement of the light harvesting in the near-infrared region, as it was shown by DRS (Fig. 5). However, based on the XPS analysis, it was clear that the Cu-CN-pl sample had undergone significant changes. Apparently, the use of plasma in the synthesis of this photocatalyst caused not only the deposition of Cu/Cu_2_O/Cu(OH)_2_ over the CN surface, but also the changes in the CN structure. A significant decrease of the content of sp^2^-bonded nitrogen did not cause the activity reduction, as in the case of CN-pl, probably because the breaking bonds served as the binding sites for Cu/Cu_2_O/Cu(OH)_2_ NPs. In that way, the plasma method led to the very intimate contact between CN and Cu/Cu_2_O/Cu(OH)_2_ NPs and enhancement of the interaction in the formed heterojunction, so the separation of charge carriers was more effective. This was further confirmed by almost identical CV curves (Fig. S5b) before and after TPC measurements, indicating material stability during illumination.

Based on all the obtained results and conclusions drawn from them, it is likely that after the irradiation of Cu-CN-pl, photogenerated e^−^ from the conduction band of CN were transferred to the composite NPs consisted of Cu in the core and the mixture of Cu_2_O/Cu(OH)_2_ on its surface. In that way, electrons can participate in reducing Cr(vi), while holes are free to react with citric acid, used as a hole scavenger, causing the improvement of the photocatalytic efficiency.

## Conclusion

In this study, we explored the synthesis of Cu-CN photocatalysts by using chemical reduction of Cu^2+^ with NaBH_4_ (Cu-CN-cr) or AP-DBD plasma discharge using Cu^2+^ in an ethanol/water mixture (Cu-CN-pl). The performances of the Cu-CN samples were tested in the photocatalytic reduction of Cr(vi) under visible light irradiation and compared with those of parent urea-derived CN and the sample CN-pl, obtained by the plasma discharge on the CN without Cu precursor.

The CN-pl sample exhibited analogous characteristics to those of the CN, with the exception of a modest loss of sp^2^-bonded nitrogen and partial breaking of aromatic rings in the CN structure, which caused slightly lower photocatalytic activity than that of CN. A comparison of the Cu-CN samples clearly demonstrates the decisive impact of synthesis method on samples properties. Specifically, the Cu-CN-cr sample exhibited the presence of Cu^+^, most likely coordinated to the N atoms. Conversely, the surface of the Cu-CN-pl sample was found to be covered with NPs of about 20 nm in size, which were identified by XPS as both Cu_2_O and Cu(OH)_2_. The presence of metallic Cu, likely in the core of NPs, was suggested according to TEM and DRS spectra. The nitrogen defects, predominantly located at the sp^2^-bonded nitrogen sites, functioned as the binding sites for Cu species, which provided homogeneous distribution of non-agglomerated NPs. It is hypothesized that, in the case of CN-pl, plasma aging led to the loss of surface activity and the healing of surface functional groups. In contrast, the formation of bonds with Cu-based NPs in Cu-CN-pl prevented these effects.

Both Cu-CN samples provided enhanced decrease in Cr(vi) concentration in the dark in comparison to CN and CN-pl, but their photocatalytic activities under the same conditions were very different. Although the absorption edge of the Cu-CN-cr was red shifted, PL peak intensity was reduced and TPC response was increased in comparison to CN, this sample exhibited the lowest efficiency, probably because photogenerated electrons were utilized for the reduction of Cu^+^ within the structure rather than Cr(vi) in the solution. Conversely, the plasma treatment resulted in significant structural transformations and deposited Cu/Cu_2_O/Cu(OH)_2_ NPs in the case of Cu-CN-pl, which resulted in the highest photocatalytic efficiency for Cr(vi) reduction, although the light absorption was not improved. The enhanced photoactivity of Cu-CN-pl is attributed to the superior interfacial contact between CN and Cu/Cu_2_O/Cu(OH)_2_ NPs within the heterojunction, which facilitates efficient electron–hole separation.

## Author contributions

Jana Petrović: conceptualization, investigation, methodology, formal analysis, writing – original draft; Anđelika Bjelajac: data curation, formal analysis, investigation, supervision, validation, writing – review & editing; Tihana Mudrinić: data curation, formal analysis, methodology; Jérôme Guillot: data curation, validation, resources; Simon Bulou: data curation, formal analysis, validation, supervision, project administration, writing – review & editing; Rada Petrović: conceptualization, methodology, writing – review and editing, supervision.

## Conflicts of interest

The authors declare that they have no known competing financial interests or personal relationships that could have appeared to influence the work reported in this paper.

## Supplementary Material

RA-016-D5RA08483K-s001

RA-016-D5RA08483K-s002

RA-016-D5RA08483K-s003

## Data Availability

The data used to support the paper are included within the article. Supplementary information (SI) is available. See DOI: https://doi.org/10.1039/d5ra08483k.
