# Supplementary figures and images for: Tuning photocatalytic activity of g-C3N4 through Cu deposition via chemical reduction and a DBD plasma method for visible-light-driven Cr(vi) reduction
Source: RSC Adv. 2026 Mar 31;16(18):16376–88. doi: 10.1039/d5ra08483k (PMC13037484; doi:10.1039/d5ra08483k)

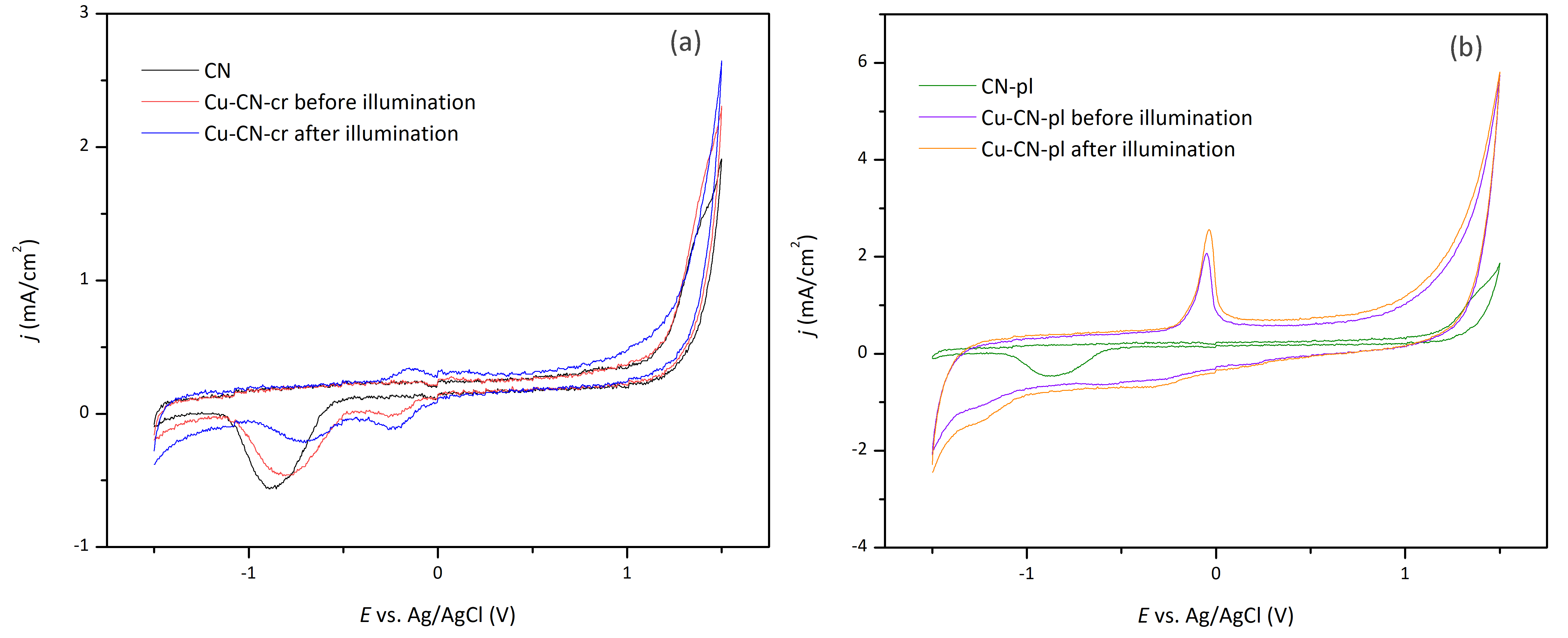

Supplement: RA-016-D5RA08483K-s002 [file RA-016-D5RA08483K-s002.zip › fig s5.tif]

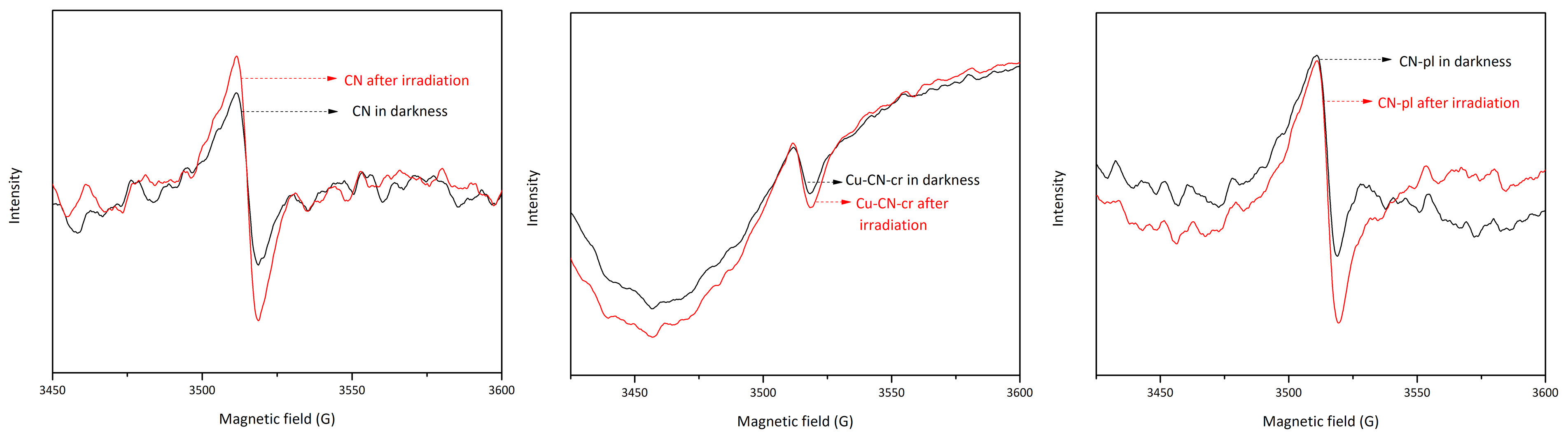

Supplement: RA-016-D5RA08483K-s002 [file RA-016-D5RA08483K-s002.zip › fig s2.tif]
