# Supplementary material for: Tuning photocatalytic activity of g-C3N4 through Cu deposition via chemical reduction and a DBD plasma method for visible-light-driven Cr(vi) reduction
Source: RSC Adv. 2026 Mar 31;16(18):16376–88. doi: 10.1039/d5ra08483k (PMC13037484; doi:10.1039/d5ra08483k)
Supplement: RA-016-D5RA08483K-s003 [file RA-016-D5RA08483K-s003.pdf]

Supplementary Information (SI) for RSC Advances.
This journal is © The Royal Society of Chemistry 2026

Supplementary Material

Tuning photocatalytic activity of g-C_3N_4 through Cu deposition via chemical reduction and DBD plasma method for visible-light-driven Cr(VI) reduction

Jana Petrović,[a] Andjelika Bjelajac,[b] Tihana Mudrinić[c], Jérôme Guillot,[b] Simon Bulou*[b] and Rada Petrović[d]

[a]Innovation Center of the Faculty of Technology and Metallurgy, Ltd, Belgrade, Serbia
[b]Luxembourg Institute of Science and Technology, Advanced Plasma and Vapor Deposition Processes Engineering, L-4362 Esch-sur-Alzette, Luxembourg
[c]University of Belgrade – Institute of Chemistry, Technology and Metallurgy – National Institute of the Republic of Serbia, Belgrade, Serbia
[d]University of Belgrade, Faculty of Technology and Metallurgy, Belgrade, Serbia

Raman spectroscopy

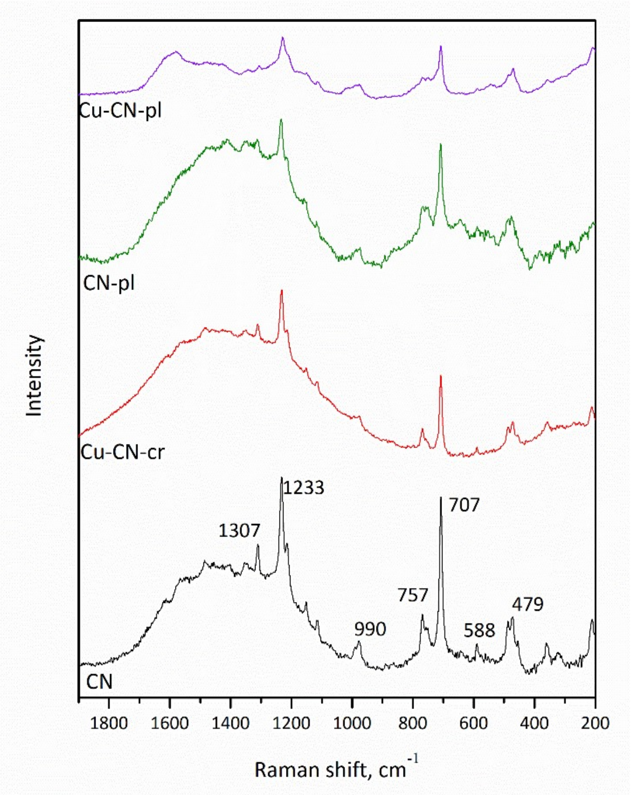

Fig. S1 Raman spectra of the CNs

EPR analysis

Table S1. Peak intensity (a. u) per 1 mg of the CN-based samples

Sample	CN	Cu-CN-cr	CN-pl	Cu-CN-pl
**Intensity - dark**	$3.30 \cdot 10^5$	$1.55 \cdot 10^5$	$4.43 \cdot 10^5$	-
**Intensity - light**	$5.07 \cdot 10^5$	$2.19 \cdot 10^5$	$5.66 \cdot 10^5$	-

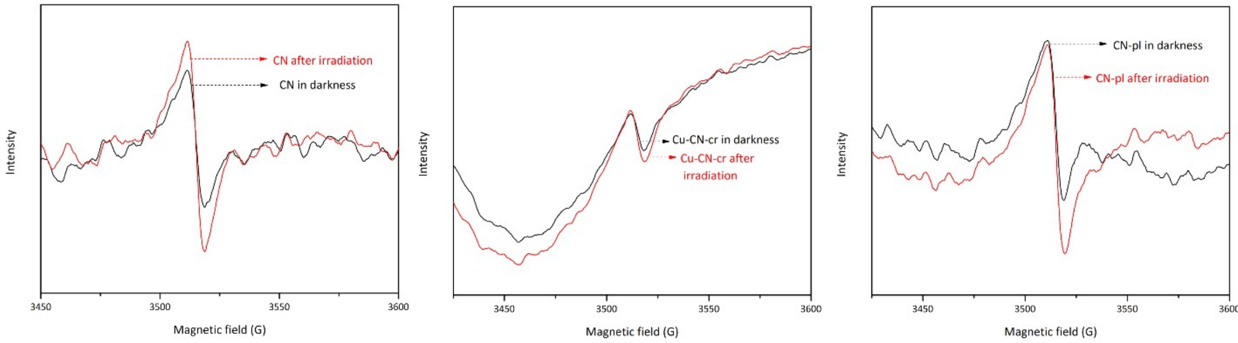

Fig. S2 EPR spectra of the CNs

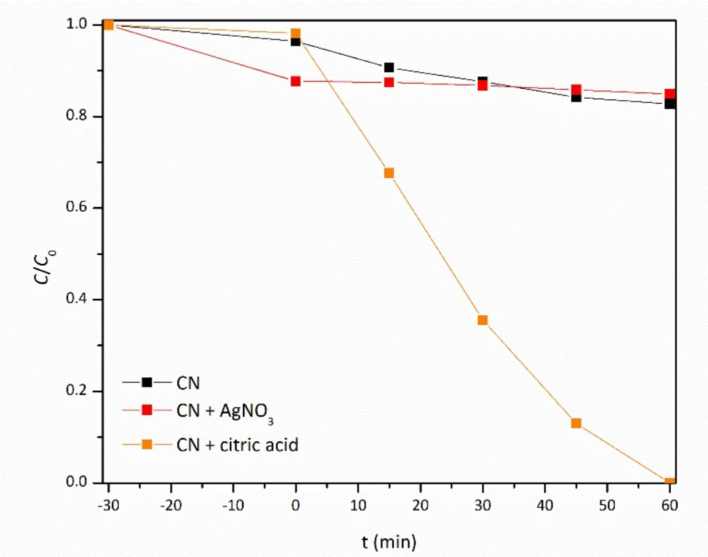

Effects of scavengers and kinetic

Fig. S3 Photocatalytic reduction of Cr(VI) under the visible irradiation with the CN

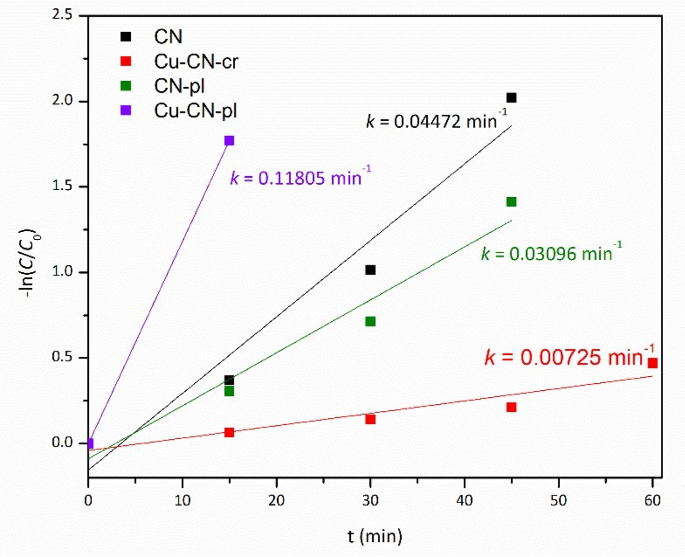

Fig. S4 Pseudo-first order kinetics plots

The photocatalytic reduction of Cr(VI) follows the pseudo-first-order equation[1]:

$$-ln\frac{C}{C_0} = k$$

Where C is the concentration of the solution at reaction time t, C_0 is the concentration of solution before the irradiation and k is rate constant.

Cyclic voltammetry (CV)

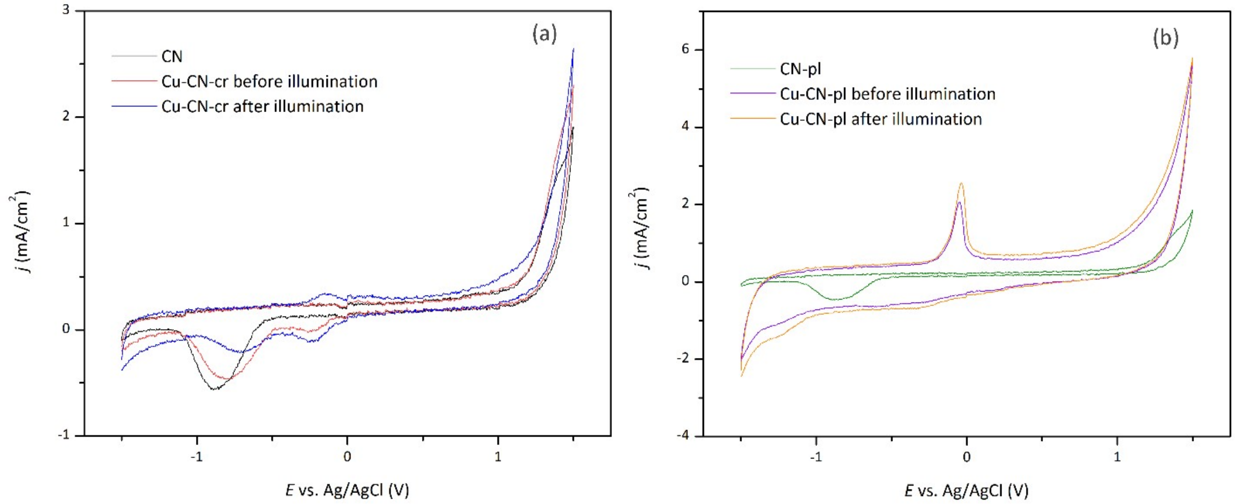

Fig. S5 CV curves of CN samples

Reference:

1 S. Liu, W. Zhang, P. Zhu, S. Zuo and H. Xia, *J. Environ. Chem. Eng.,* 2021, **9**, 105879.
